# Mr. Ring, on Vaccination

**Published:** 1806-07

**Authors:** John Ring

**Affiliations:** New Street, Hanover Square


### To the Editors of the Medical and Physical Journal.

GENTLEMEN,

IN the Sherborne Mercury for May 5, is the following correspondence, which, I doubt not, will prove interesting to your readers.

### "THE RAVAGES OF THE SMALL-POX.

"To Dr. CREASER, *Bath.*

"DEAR SIR,

"The small-pox is raging with unexampled mortality, in consequence of inoculation, in the neighbourhood of Stogursey; and, among the few who have been prevailed on to submit to this dreadful disease, I am informed that eight or nine have fallen victims.

"You will startle at hearing, that professional men dip their lancets in this cruel poison; and communicate an uncontroulable disease to their fellow creatures. You will be still more astonished at finding, that the minds of some respectable inhabitants are still warped by prejudice against the cow-pock, as a preventive of this scourge of the human race; but your wonder will cease when you learn, that a story is in circulation, and believed by many people, of a lady who has been corresponding with the immortal Jenner, whose opinion she represented as repugnant to the reputation of the cow-pock; and this as freely as may be, declaring that Dr. Jenner himself has no great opinion of the practice.

"Is this possible? Are these the sentiments of that great man? If so, he must be strangely altered since last summer; when I had the pleasure of meeting you at his hospitable mansion. However, it has had a considerable effect on the minds of the ignorant and unwary; and the example of this lady, who has had her own children inoculated for the small-pox, is an additional motive to induce others to follow it.

"I have inoculated two thousand and seventeen for the cow-pock, without a single instance of failure, or any alarming occurrence: and there can be no doubt, where medical men are attentive, and procure proper vaccine virus, of its proving the greatest blessing ever bestowed by heaven on man.

"But I believe there are some inveterate enemies of this practice through ignorance, and others through obstinacy; who will not suffer any evidence, however strong, to impress their minds with conviction; and who will not be guided by any experience, however extensive.—I trust you will communicate to our friend this report concerning him, as I am not certain of his address at this time; and I will thank you for an answer, as soon as you receive his.

I am, &c.

*Wiveliscombe Dispensary,* HENRY SULLY."
*April* 7, 1806.

---

"To HENRY SULLY, Esq.

"DEAR SIR,

"In consequence of your information, that a report had been industriously circulated in the vicinity of Stogursey, purporting that Dr. Jenner had abandoned his opinion concering vaccination, I addressed a letter to him on the subject; and I am authorised by him to declare, that this story is totally unfounded; that he never, either in conversation or correspondence, gave the smallest hint of any alteration of opinion on the subject, but that, on the contrary, every day produces new evidence of the security and advantages of vaccination, both here and in foreign countries.

"Had I not had ample experience, for many years, of the industry and malignity of the opponents of vaccination, I should wonder at the existence of so bare-faced a fabrication as that in question. A very small portion of the active efforts, which are made by the enemies of mankind, to perpetuate one of its greatest scourges, the small-pox, would, if properly directed, be sufficient for its extinction.

"How base and criminal is this conduct! and how consistent, in point of atrocity, are the means with the end! I have the pleasure to communicate to you the thanks of the Royal Somerset Jennerian Society, for your successful endeavours, in the prosecution of their plan; and I am also commissioned by our friend Dr. Jenner, to present you with his acknowledgments for the same.

I am, &c.

THOMAS CREASER."

*Bath, April* 13, 1806.

This

This correspondence is an incontestible proof, in addition to many others which I have already laid before the public, that the opponents of vaccination do not hesitate to invent, as well as to circulate, the most gross and impudent falsehoods. This has been their practice, from the earliest periods of vaccination, to the present hour; and these falsehoods they have propagated, and still propagate, in every form,

——————— ne quid inausum,
Aut intentatum, scelerisve dolive relinquant.

Among others, of a similar kind to the foregoing, are the following, which I published in the year 1801; and which are a sufficient refutation of that other bare-faced and impudent falsehood, so often advanced in the face of day, and already so often refuted, namely, that the friends of vaccination have never instituted any inquiry, in order to ascertain the truth. *See Treatise on the Cow-pox, p. 269.*

A man, whose wife had the confluent small-pox, requested Mr. Kelson of Sevenoaks, to inoculate him for the cow-pock, with which he complied, and desired to see him again in three days; but the man did not come again till after the expiration of a week, when he was inoculated again. This was on the tenth day, from the time when the small-pox had appeared on his wife.—The second inoculation took place; but too late to prevent the small-pox. It was, however, mitigated; for Mr. Kelson remarked, that the pustules turned much sooner than he had ever known them in any other instance, where the eruption was equally copious.

This case, however, gave rise to several false reports, which were at that time, as others of a similar kind have been since, countenanced by a despicable cabal; who have undesignedly been instrumental in promoting a cause which they meant to injure; and have reason to repent the discussion they provoked.

Then, as well as now, it will appear from the same publication, p. 270, they propagated a report that Dr. Jenner himself entertained a doubt of the efficacy of vaccination; and that his own servant, whom he vaccinated, had since died of the small-pox.—This report having been industriously circulated, and even mentioned in different medical societies, with some degree of confidence, I wrote to Dr. Jenner, in order to know what degree of credit it was entitled to; and received the following answer.

"The whole of the assertion you heard at the Medical Society, respecting my entertaining doubts of the efficacy of the cow-pox in preventing the small-pox, is

*entirely*

*entirely false*; and must have been invented by some malevolent person, with a base design. The idea I ever entertained of the security of the patient, has been strengthened by my late experiments. Many of those, who were inoculated with variolous matter, have again been subjected to the same test. Some have had sheets wrapped round them, in which those had lain, who had laboured under full burdens of the small-pox. Some have had the matter thrust up their nostrils; and others have been put into beds with those, who had the small-pox in its highest state of infection; but they all resisted its action."

Such were the rumours at that period, and such were the refutations of those rumours; yet, among the opponents of vaccination, persons have not been wanting, who assert, with the most unblushing effrontery, that no inquiry has been made into the subject.

The following is another specimen of the falsehoods, which are even now in circulation. A woman at Kensington, whose children had the itch, lately spread a report, that they had an eruption in consequence of the cow-pock. Having heard this report from a gentleman in Hanover Square, who is a member of parliament, and whose opinion must have great weight with the public, I inquired into it; and found it totally destitute of foundation. The children had indeed an eruption; but it was neither more nor less than the itch; and the mother confessed, that she had procured ointments from Mr. Faithhorn for this very disease. In order to conceal its real nature, she not only asserted, that her children had eruptions from the cow-pock, while she treated them as the itch; but also denied that her other children had the same disorder, which, on examination, I discovered to be a gross and palpable falsehood.

In the publication before quoted, p. 82, I long ago observed, "that such persons as had not seen the works of Jenner, nor examined what was written in favour of vaccination, had amused themselves with a newspaper, or a magazine; and poisoned their minds with the falsehoods there inserted, falsehoods known to be such by their own authors." I also observed, "that some of the unfavourable reports raised against the practice, were *palpable forgeries*; that the name of the person, perhaps, was mentioned, but not the residence; that no signature was affixed, *or only such as would injure the best cause.*" The same course has still been pursued, and the same practice prevailed, from that period to the present hour.

In

In the same publication, p. 36, I remarked, that were I to recite all the arguments brought forward by Dr. Jenner alone, to prove that vaccine virus, in its perfect state, is a security against the small-pox, no unprejudiced reader would deny that I had sufficiently established my position;" but I also remarked, *that "artful and designing men had deluded the public by false representations;"* and "that one of them, when repeatedly called on, actually refused to give such evidence of the truth of his assertions, as he had publicly pledged himself to give; and shrunk from all inquiry."

I also stated, that some few respectable practitioners had been deluded by false reports, and expressed their apprehensions in a becoming manner; but that there are certain persons *in the lower order of the profession* who rise up in arms against it,—

> Omnigenûmque deûm monstra, et latrator Anubis,
> Contra Neptunum et Venerem, contraque Minervam,
> Tela tenent."

This was published in the year 1801, long before the question was agitated in parliament, yet it has been asserted again and again, with the most unblushing effrontery, by the opponents of vaccination, that *the learned Mr. Goldson* was the only person, except Dr. Moseley, who opposed the practice till that time. So ignorant are those gentlemen of what has been done by their own fraternity; or so desirous are they to forget, and that *the world* should also forget, *who* they are, and *what* they have done.

With regard to the forgeries against vaccination, I have adduced authorities on that head, in my answer to Doctor Moseley, in addition to those which I long ago adduced in my other publications. When I printed the account of the cases fabricated against the practice, by the second great antivaccinist, that champion of falsehood was still living, and still publishing other forgeries in this and other journals. The manner in which he at length terminated his unfortunate career, will, it is hoped, prove a warning to others, who are now treading in his steps.

These gentlemen are always pretending, though they know it to be false, that the friends of vaccination have never instituted any inquiry into the merits of the practice. In opposition to this bold and unfounded assertion, I shall here subjoin the free and unsolicited testimony of an author, who not being engaged in the busy scene of this metropolis, nor apparently having any particular interest rest in the issue of the contest, is more likely to be impartial than certain gentlemen who have lately given their opinions on the subject. This testimony is extracted from a publication called, "A Concise Treatise on the Progress of Medicine, being the Substance of the Thurstonian Oration, delivered at Cambridge in 1802, by W. H. Williams, M. B. of Gonville and Caius College, Physician to the Ipswich Dispensary."

"I now come to the last, and greatest discovery of this, or perhaps any, splendid æra of medical improvement. I contemplate a blessing, which extends its benign influence from the palace to the cottage; which, though wealth might rejoice to purchase, is accessible to poverty; without the loss, or suspension, of its useful labour. This is the happy introduction of the vaccine inoculation, to supersede the prevalence, and extirpate, if possible, the existence, of that afflicting scourge the small-pox. To describe the simplicity of its inoculation, the safety of the disease, and the innocence of its effects, were superfluous, since each is become a matter of general notoriety. The opposition it met with, from the laudable caution of medical men, and the fears of the timid and affectionate parent, begin to subside; and indeed, may be said to disappear.

"Inaccurate statements of cases, and histories of the disease, where this inoculation was supposed to fail, or its effects to have disproved the allegations of its patrons and advocates, have, at various times, appeared; but *a candid and able investigation of those cases which seemed of importance, has been always instituted by the promoters of the vaccine inoculation, with a zeal that bespoke philanthropy, and an alacrity that manifested conscious rectitude.* The result of the investigation *has answered their best wishes, and vindicated this noble discovery, by an easy detection of the blunders, incapacity, or oversight of those, in whose hands the cases were said to have failed.*

"Various attempts have been made, to deprive Doctor Jenner of the honour of being the discoverer of vaccine inoculation; as, however, none deny him the merit of first making the discovery of its universal utility, and, in the words of Dr. Johnson, as *He only discovers who proves,* such attempts will be despised as impotent, or condemned as invidious.

"The first city in the world, with its chief magistrate at its head, aided and animated by the first nobility, and eminent commoners in the kingdom, has stood forwards the the patron of the vaccine inoculation. The British legislature has expressed its mutually honourable approbation, by bestowing on its author the munificent liberality of a great nation.

"If life be dear, if it be a blessing to preserve the index of the mind, and the feelings of the heart, from being impaired or blotted out of the countenance,—if one dread blow from the dagger of death be foiled, and a shield added to the feeble arm of man,—if these be objects of admiration and gratitude, we must venerate the victory gained over the ravages of the small-pox by Dr. Jenner. How grateful is the office to speak the public gratitude!—how exalted the happiness to deserve it!

"Can I forget, then, his memory who pointed out to us so excellent a service? No! addressing ourselves gratefully to the manes of the venerable Dr. Caius, let us exclaim,

"O virum utilem sibi, suis, reipublicæ, et humano generi!"

Such is the voluntary tribute of applause offered to Dr. Jenner, and the friends of his valuable discovery, by an unbiassed author, whose testimony far outweighs that of all the self-interested medical opponents of vaccination in this metropolis; where, as Dr. Rowley justly observes, men are *jealous competitors for practice.*

To this testimony we may add that of Mr. Moore; who says, "The misrepresentations, and mistakes, of the anti-vaccinists, must be answered in the mass; it would be endless to refute them individually. The *misrepresentations are most numerous.* It is *to be hoped* that they are not formed with a bad design; but proceed from the same beneficent principle, which, in the early ages, so often occasioned the invention of pious frauds.

"With regard to *mistakes,* the impossibility of always avoiding them *in the small-pox,* has already been noticed; and, in some few instances, the same difficulty occurs *in the cow-pock.* All the peculiarities of this curious complaint were not to be detected at once: it was not to be expected, that within the first two or three years, the art of vaccination should be brought to perfection. *Not the slightest dependence,* however, *can be placed upon any one of Dr. Rowley's cases;* for he fairly owns, that where the small-pox followed vaccination, *he thought it quite irrelative to the question, to inquire whether the cow-pock took, or whether it was genuine or spurious.* Dr. Rowley must forgive me; but *he is evidently too much prepossessed, to be a fit person to investigate this subject.* It seems even probable, bable, that when an eruption occurred, he would think it equally irrelative to inquire, whether it was really the small-pox, or some other eruption.

"It is superfluous to dwell longer on the subject; enough has been said, to repel all the objections hitherto brought against vaccination. Errors may possibly have occurred in the practice of this inoculation, as well as in that of the small-pox. They are inseparable from the exercise of every human art; but they constitute no objection to the principle of the practice, which is established in truth. A little more time will settle every disputed point; diffuse the knowledge that is acquired; and dispel the prejudices of *the inferior practitioners in medicine*, and of the vulgar.

"An improved method of treatment has rendered the small-pox less destructive than it formerly was; and inoculation commonly renders it safe to the person inoculated; yet it is admitted by all *eminent* and *candid* physicians, that a part of those who are inoculated, die. The proportion of deaths, under inoculation, it is difficult to ascertain; no practitioner, for obvious reasons, being willing to acknowledge *the whole truth* on this delicate point. All writers however agree in this, that *the confluent small-pox* is sometimes produced by *inoculation*. They likewise mention *epileptic fits* as *a frequent occurrence*; and there is *no man, whose testimony is worth listening to, who can dare to assert, that the confluent small-pox, and epileptic fits, are not very alarming circumstances.* As these are not uncommon, some, and especially the weakly, must sink under them.

"The small-pox then, notwithstanding the mitigation of the disease by inoculation, and the present improved state of medicine, continues to be a dreadful evil. It considerably diminishes the population of the world; afflicts a portion of the remainder with blindness, and excites the scrofula into action.

"It is to be hoped, that the opponents of vaccination will seriously reflect on what they are doing; and abate a little of *their cruel zeal.* Dr. Rowley's certainly carried him too far, when he brought *religion* to his aid; which has no more to do with vaccination, than with inoculation; or any other operation of surgery."

Mr. Moore afterwards observes, that Dr. Rowley has bewildered himself in a chaos of medical theology. "I shall therefore," says he, "take my leave of him; having, by these specimens, given a sufficient notion of *his intel-*

*lectual*

*lectual powers*, and *those of his coadjutors*. It may fairly be inferred, that they are *not of that transcendant kind*, as to entitle them to treat the united medical professors, and distinguished practitioners, as far inferior to them, and even as mad men.

Mr. Moore is of opinion, that although *the authority* of the opponents of vaccination, in spite of all their vehemence, will have little weight with the enlightened part of the public; their *arguments* and *observations* will probably hasten a decision. It requires no spirit of prophecy to foretel, that this decision will be against them, as it was in parliament; where, it was universally allowed, nothing could strengthen the evidence in favour of Dr. Jenner's petition more, than the witnesses that appeared in its favour, *except the witnesses that appeared against it.*

Mr. Moore observes, that our enemies, the French, have adopted vaccination with all the ardor natural to that enthusiastic people; who are anxious to promote their population, augment the number of their soldiers and sailors, and extend their power.—"And shall *we*, who find *so much difficulty in recruiting our military force*, obstinately continue to perpetuate a mortal disease, by inoculating for the small-pox, and refuse to adopt universally, a discovery made at home?"

" It is the fate of all *great discoveries*, to be opposed by *little minds*. When the immortal Harvey discovered the circulation of the blood, all *distinguished* medical men understood his demonstration; and received it with rapture. But his discovery was opposed and ridiculed by *that part of the faculty*, who were incapable of comprehending it.

" When the inoculation of the small-pox was introduced into this country, it was likewise opposed by *the dregs of the profession;* and *the same objections*, accompanied with *the same species of proof*, were brought against it, as are now brought against vaccination. It was *falsely pretended*, that *humours* were thus introduced into the body; and that those who were inoculated were not secure from having the disease again; and lists of cases were published in support of these objections. It is to be hoped, that the opponents of vaccination will cease in time; and escape, by a candid recantation, from the mortifying reproach of being classed with those, who opposed the two great discoveries mentioned above.

" But the accomplishment of this object, which is so highly interesting, not only to the happiness of individuals, but also to the prosperity of the state, ought not to be left left to the slow progress of reason against prejudice.—The legislature should interfere, in order to protect the people against those, who scatter a mortal contagion through the land."

As to the religious scruples, which the enemies of vaccination pretend to feel; no one who is acquainted with the *birth, parentage,* and *education, life, character* and *behavior,* of any one of them, can for a moment believe such scruples to be sincere.—They are a mere *ad captandum,* an artifice calculated to seduce the vulgar. Mr. Moore remarks, that a religious scruple has been the chief obstacle of inoculation in general. "*All enlightened Christians,*" says he, "*saw the folly of this objection.* There is no precept in the gospel, to prohibit us from preserving ourselves from disease and death. As soon, therefore, as it was proved, that inoculation contributed to save our lives, *men of sense,* from duty, as well as from inclination, adopted it; and for the same reason, now that they are convinced vaccination is safer than the inoculation of the small-pox, they give the preference to the former."

Mr. Moore observes, that those gentlemen who doubted, or, at least, *affected to doubt,* the immense multitude of well authenticated facts favouring the practice, are credulous to excess of every obscure case of a contrary tendency. He observes, that this extraordinary discovery was quickly conveyed to every part of the globe, where letters have penetrated; that it was not a mere rumour, swallowed and diffused by the credulous populace; but propagated by *competent judges,*—*learned professors,*—*sagacious physicians,*—and *skilful surgeons;*—men who were fully aware of the danger of trusting to a plausible theory,—and even to the fallacies resulting from the representation of facts.

He adds, "Age, experience, and reason, make such men slow in adopting innovations. They trusted little to the trials of others; they repeated the experiments themselves.—The same effects invariably occurred; conviction followed.

"The ingenious, the prudent, the profound, in France, Spain, Italy, Germany, the Northern Nations, and the Indies, eagerly adopted this splendid discovery.—Even the prejudiced Turk has been tempted, in some degree, to abandon his confidence in predestination; and to inoculate with the cow-pock. Who, then, are those *confident* men, who venture to come in competition with such a conjunction of learning and intelligence? who have the boldness ness to arraign all that are eminent in medicine, in every part of the civilized world,—accusing them, not only of committing a gross error, but of madness,—who exalt themselves as of higher authority, more profound reasoners, and more exact observers of Nature? I do not pretend to measure the capacities of such *extraordinary men*; their own works are the proper criteria, by which their claims to this stupendous merit must be decided."

Mr. Moore accordingly examines their works, and proves them to be the mere scum of learning, and dregs of wit; an outrage against decency,—an insult to common sense,—and a conspiracy against the welfare and happiness of mankind.—With respect to failures, or at least apparent failures, he justly remarks, that they occasionally happen in the small-pox as well as the cow-pock; and with respect to diseases subsequent to vaccination he observes, that the cow is healthy, that the most diseased animal in Nature is man, and that the cow is indebted for this disease to the contact of the hand of man.

After this testimony, none of my readers will be surprised at finding, that other reports, lately brought forward, are, like the former, only brought forward to be refuted. Among these are the reports of failures at Bristol, in the practice of Mr. Henry Jenner. I have now before me, a letter from him to Dr. Jenner, in which he says, "I have vaccinated thousands; but no person, vaccinated by me, was ever afterwards infected with the small-pox, either by inoculation, or exposure to its contagion.—Vast numbers of my vaccinated patients were inoculated with variolous matter, at distant periods, and many of them put to the test, every way, in which it was thought possible to infect them; but such confidence did I place in its security, that my own children were several times exposed to its contagion, years after the cow-pock, by wearing lint dipped in variolous matter up their nostrils. They have also remained with persons covered with the confluent small-pox, in a small room, where the smell was intolerable; and many times been inoculated with the most active virus; but in vain. "I have seen some of them lying in the same bed with persons covered with the small-pox, and a child at the breast of its mother labouring under that disease; but they all resisted infection."

I have also seen the copy of a letter from a gentleman of Bristol to the Reverend Mr. Jenner, in which he requests Mr. Jenner to lend him a certain publication; declaring, that he was unwilling to purchase it, lest, by promoting the sale of the book, he should *encourage the rogue*

*to go on.* He says, a gentleman whom he waited on, to inquire into the case of his child, is astonished at the effrontery of the man, who has roundly asserted such a falsehood; nor will he believe it, but from ocular demonstration.—Many people are of opinion, that there ought to be a law to punish *medical forgeries*; but surely it is a sufficient punishment, to bear the compunctions of a guilty conscience, the curse of parents, and the contempt and indignation of every honest man.

The antivaccinists will never be tired with telling the world, that the friends of the practice, in their zeal, and their philanthropy, may perhaps have been rather too sanguine in their hopes, and rather too precipitate in their conclusions. But they have never yet proved, that the friends of vaccination have wilfully deceived the public. Happy would it be for the antivaccinists, if they could lay their hands upon their hearts, and say the same. I would recommend to those geutlemen to pluck the beams from their own eyes, in order that they may see the more clearly, how to pluck the motes from the eyes of their brethren. I would also advise them to read Dr. Mac Donald's answer to Mr. Goldson, in this Journal for October, 1804, which concludes in the following words:

"He who, from a zeal for the discovery, should suffer his eyes to be shut against conviction, and attempt to conceal its failures, would indeed commit an act beneath the dignity of the profession; *but he who imposes on the ignorant, under the mask of candour and moderation,—who spreads vain alarms, and provokes controversy upon a subject, in which he must so sensibly feel his own deficiency, is guilty of a deed far more beneath the dignity of the profession; and far more unbecoming one, who is intrusted with the welfare and happiness of the public.*"

At the late anniversary of Dr. Jenner's birth-day, an Address was presented to the Royal Jennerian Society, by Mr. Carey, Author of "the Pleasures of Nature, the Reign of Fancy, and other poems, in which he alludes to the illiberal and interested opposition which has been raised against vaccination. I shall quote one passage; in which I have taken the liberty to make a slight alteration in the diction, without altering the sentiment.

"Ye gen'rous few, who on the labour smile,
And share alike the glory and the toil,
Proceed, the sum of sorrow to assuage,
Proceed,—nor fear detraction's *venal* rage.
The happy plant you rear, if tempest blown,
Shall friendly shelter find beneath the throne."

His

His Royal Highness the Duke of York, honoured the meeting with his presence, and informed the company, that from the first establishment of the Society, he had been a warm friend to the institution, from a firm conviction that it would be of unspeakable benefit to mankind. He assured them, that no one can more sincerely wish success to the Society, than he has done. He expressed his regret, that the *misrepresentations* of certain *interested* individuals has in some measure damped the ardour of the friends of vaccination, and checked their exertions; but he had no doubt we should soon evince to the world, that the advantages resulting from the practice are above all opposition; and that its beneficial effects will speedily be extended to every part of the globe.

At the same meeting, Dr. Lettsom lamented the prevalence of variolous inoculation, and the privilege which certain sordid and mercenary practitioners still enjoy,—of doing mischief.—He observed, that some medical *gentlemen* in this city, have circulated many false reports against vaccination, and thereby renewed the inoculation of the small-pox; by which a considerable number of lives have been lost. He nevertheless felt a confidence, that when the virtuous exertions of the Society, who have already vaccinated about twenty thousand persons, are generally known, their prospects will brighten; and they will at length be enabled to triumph over all the misrepresentations, and interested opposition, of their insidious and inveterate foes.

Dr. Jenner remarked, that while vaccination was making a rapid progress in other parts of the world, several medical practitioners in the metropolis have ungenerously circulated misrepresentations respecting this salutary practice; and endeavoured to defeat its purpose.—He observed, that various attempts have been made to contaminate the public mind, by *low* and *delusive pamphlets, circulated with industry, and replete with falsehoods.* He himself has been accused of inoculating, without success, children of whom he had never heard, in a village which he had never seen. He declared that similar falsehoods have been fabricated, and circulated, concerning the practice of his nephew, Mr. Henry Jenner. He flattered himself, however, that the good sense of the people of England would soon rise superior to such wicked arts, and mischievous attempts; and that vaccination would at length become as general in this country, where it was first discovered, as it is in other parts of the world, where it has since been adopted.

The Reverend Rowland Hill informed the Society, that, deeply impressed with a conviction of the manifold blessings, with which a general diffusion of vaccination must benefit mankind, he had paid the most profound attention to the practice, and given it the most extensive encouragement. He had vaccinated upwards of five thousand persons, without being able to discover a single failure. He had also made inquiries into the success attending it, in all those parts of the country which he has occasionally visited; and uniformly received the most favourable and flattering accounts of the good effects resulting from it, wherever it has been introduced.

"He said, it is calculated that forty thousand persons annually die of the small-pox in this kingdom; and expressed his opinion, that *it would well become the Legislature to inquire, how far men ought to have the liberty of killing themselves, and each other.* This sentiment he had most sensibly felt; and in order to remedy the evil, as far as was in his power, he had warmly recommended the practice in his chapel; in consequence of which, upwards of four thousand more have been vaccinated in that neighbourhood.

He said, he was astonished to find, that there should be men of some education, who were so perverse, as to endeavour to impede the progress of a practice, from which so many benefits and blessings to the human race must result; but he justly remarked, that they are *men who live by disease.* He said, it is strange, but to those who know the parties it is not strange, that they should circulate *the grossest misrepresentations and falsehoods,* through the medium of low and illiberal pamphlets, in order to raise themselves from obscurity.

The Earl of Egremont also bore testimony to the efficacy and utility of Dr. Jenner's discovery, several thousands having been vaccinated at Petworth; where, from an early period, the practice has been honoured with his Lordship's most zealous patronage and support.

In some respects, the account of the meeting, published in the daily prints, is inaccurate; particularly when it is stated, that out of the numbers vaccinated by the Jennerian Society, and by Dr. Reyss of Poland, some had died. It was not admitted, that any one had died of vaccination, either in the practice of the Society, or of Dr. Reyss. Some particulars of his letter to Dr. Jenner, of which I read read a translation to the Society at their late meeting, having been misunderstood, I shall endeavour, in this and other channels, to rectify the mistakes which have occurred.

*The following is a Copy of the original Letter.*

"Johannes Reyss ad Illustrem Magnificum Edovardum Jennerum, &c. &c. &c. Variolarum mortiferarum Eradicatorem præclarum.

"Nuper mihi, a quodam amico, scyphum argenteum comparavi, cui casu Dantisci in venditione publica emptus venerat. Diligentius artem et operam intuenti, cum primum nomen ibi Jenneri, sub die 9 Martii Anni 1745, excusum repererim, mox inde summum lætitæ gradum expertus sum; serioque intentus, ut quod aliquo fortassis eventu é gazophylacio Ipsius dignissimorum Progenitorum alienatum fuerit, quantocius reverti queat.

"Attamen interest votis meis plenissimo cumulandis gaudio, ut exiguum hocce animi mei erga Te, vir præclarissime, documentum, faustissimo natalitiorum tuorum die, nempe 17 Maii, in loco ipso sistatur, quo, communis lætitiæ causa, alma Tuorum, seu potius humanitatis Amicorum Societas, Te diu incolumem, longævis temporibus sospitem, hilarissima inter convivia, circumeunte nominis tui amplitudine insignito poculo, per vices alternas proclamare et extollere poterit.

"Liceat ergo te alloqui, Clarissime Domine, verbis, quibus totius quasi Orbis consensus nomen tuum resonat, ac remotissima posteritas suum benefactorem profitebetur. Accipe itaque specimina magni Nominis, et honoris quem adeptus es, ac simul pignus indubitabile laudis longe lateque diffusæ; meique etiam memor esto, qui licet immensa a Te locorum distantia dissitus sim, verumtamen sincerissimo affectu, ardoreque ejusdem exercendi Instituti, tibi Vir Clarissime! copulari desidero.

"Non dedignabitur nihilominus magnitudo Tua exordia ac progressus laboris mei in vaccinatione hic allaturos iri, quod cordi suo humanitatis Zelo succenso perjucundum fore minime dubito. Prima itaque mihi, anno 1799, notitia fuit, de felici inventione Vaccinæ per Illustrem Magnificum Dominum, ac statim flagrans desiderio, in tam salutifera methodo certiorem me reddendi, omnibus conatibus ampliorem instructionem sibi acquirendam putavi, donec anno 1800, luci publicæ apparuerit Illustris Magni-

 fici fici Domini de Vaccinatione opus præstantissimum, ex Anglico idiomate in Germanicum versum, operâ D. de Kin, Lipsiæ prelo mandatum; simul etiam studui, ut quantocius aptam Vaccinæ materiam mihi procurarem, cujus tandem haud facile ex Londino per Dantiscum potitus sum.

"His igitur auxiliis adjutus, tempore autumnali, 1801, jam multum provecto, inoculare incepi octo infantes; cujus operationis gratia, cum mihi plenissime utilitatem et veritatem rei compertam habuerim, materiam pro anno subsequenti, 1802, diligenter asservavi.

"Interea D. La Fontaine, Vir in re literaria benemeritus, coepit typis Varsaviensibus mandari, ita dictum, *Diarium Sanitatis*: quo in opere multum satagebat, ut sinistræ de vaccinatione nostra opiniones propagarentur, et tam innocentissima ars, quasi in primis incunabulis suffocaretur. Ipsius hæc præjudicia, fortasse ex aliquibus auctoribus Berolinensibus deprompta, et Polonicâ linguâ exposita, vix dici possit quanta obstacula et impedimenta progressui ulteriori Vaccinationis posuere. Avidiùs ergo curam omnem, et vires meas impendi ad id, ut errores his in regnis contra vaccinationem præconceptos, ex animis evellerem. Sciens vero plus inesse fidei praxi, quam scriptis et altercationibus literariis, huic operi accinxi me anno 1802, a die 14 Junii, per provinciam, suo sumptu; ac parvulis pauperum 1433 vaccinam inoculavi; solvendo etiam aliis, ut mutuo sese adhortarentur proposito præmio, inter grassantem omni ex parte luem mortalem variolarum naturalium.

"Testem hujus rei ac operis provoco Novalias seu Gazettas Varsavienses; quibus inscribi voluit hoc, testimonii loco, Synagoga Judæorum oppidi Tarnobrzeg, ubi de sexaginta, qui a parentibus inoculationi subducti erant, viginti mortui, duo excæcati sunt; de septuaginta vero et uno, quibus inoculavi, licetsi insimul dormientes, manducantes, et ludentes fuerint, nequidem unus infectus aut mortuus est.

"Ad annum itaque 1803, sub dato 3 Martii, exposui publico operis mei præstiti ad annum 1802 notitiam; quæ in Varsaviensibus, æquè Cracoviensibus novaliis exposita, id perfecerat in animis hominum, præsertim rudiorum, ut certatim ad me concurrerent, una cum prole inoculationi traditâ. Jam vero, anno 1803, Sacra Cæsarea Majestas, publico suo patentali, in toto regno inoculandos mandavit infantes. Huic saluberrimæ dispositioni satisfaciendæ, unam sectionem pro mei personâ accepi, ac inibi inoculavi infantibus 1214; in anno 1804, infantibus 1345. Summa ma triennalis inoculationis, tantum per menses quinque sibi succedaneos, incipiendo a Maio, usque ad Octobrem, 4000 personarum; quod et Regimini authenticis documentis comprobavi, ejusque beneplacitum lucratus sum.

"Nunc vero fateor ingenue, nil tam cordi meo esse, vir clarissime, quam aggregari Instituto tuo; innotuit enim mihi, etiam extraneum hujus Instituti membrum posse fieri dummodo in hoc strenuam navaverit operam. Confido ergo benevolentiæ tuæ, illustris magnifice Domine, me quoque posse concedi participem fieri celeberrimi Instituti; quod humillime pro mei persona exoro; et hanc singularem gratiam, per favorabiles literas illustris magnifici Domini, cupidissime expectabo. Interim pro Instituto submitto 10 aureos nummos, sive 5 guineas; quas enixe suscipere rogo.

"Ex quo distantia regni, marisque incommoda, impediunt animum meum ad personam tui, illustris magnifice Domine, aliquando conspiciendum; cuperem minimum saltem effigie Ipsius frui, si ita placebit me ipsâ condonari. Frustulum quoque panni coloris hujus, in quo plurimum sibi complaces, vir nobilissime, in tesseram grati animi tui affectuose susciperem. Vellem etenim Jenneriano Nomini publicum gratum facere, in his qui ejusmodi coloris vestitu ipsâ die Natalitiorum Tuorum uniuscujuscunque anni usi fuerint, ac Instituto tuo promptas dederint manus.

"Dies ista solemnis mihi semper existet, quousque vitæ hujus perfruar aurâ. Jam nihil superest, quam ut, Vir, Clarissime, benevaleas. Hæc optat

Illustris Magnifici Domini
Cliens ac Servus humillimus,
Johannes Reyss.

"Dabam Machovii in Gallitia Orientali, prope Sandamiriam, in Circulo Rzescoviensi.
Die 8va *Aprilis*, 1805."

This letter of Dr. Reyss shews in what estimation Dr. Jenner's discovery is held by learned and enlightened foreigners. It is an interesting document, and ought to be generally understood. I shall therefore subjoin a translation, for the sake of the members of the Anti-vaccinarian Society; who, being unable to write *English*, cannot be expected to understand *Latin*. *It would be Greek to them.*

 "John

"John Reyss to the great and illustrious Edw. Jenner &c. &c. &c. the celebrated Exterminator of that fatal Disease, the Small-pox.

"I lately procured from a friend a silver vase, purchased at a public sale at Dantzic. When I examined its form and workmanship, and found engraved on it the name of '*Jenner, the* 9*th of March*, 1745,' I instantly experienced the greatest pleasure; and seriously resolved, that what had probably been alienated from the repository of his very worthy ancestors, by some unfortunate event, should be restored as soon as possible.

"Nothing is wanting, to crown my wishes, and to complete my joy, but that, on the 17th of May, the auspicious day of your birth, this small token of my esteem for you, most excellent Sir, should be placed, where, in a festive and convivial hour, as the goblet, decorated with the glory of your name, goes round, the benevolent Society of your friends, or rather the friends of humanity, may alternately drink your health, long life, and prosperity,—with loud acclammations and shouts of applause.

"Let me therefore address you, Sir, in those words, in which the whole world, as it were, with one consent, resounds your praise; and latest posterity shall acknowledge you their benefactor. Accept these testimonials of the great honour and renown you have acquired, and a sure pledge of your wide-extended fame; and bear me also in remembrance, who, although far remote in space, am nevertheless attached to you by the most sincere affection; and burn with an ardent desire to be connected with you in the exercise of your art, and in promoting the object of your institution.

"Permit me, Sir, to lay before you an account of the rise and progress of my labours in vaccination; which, I have not the least doubt, will be extremely grateful to a heart like yours, which is so zealous in the cause of humanity.—In the year 1799, I first received notice of the happy discovery of the great and illustrious Jenner; and instantly feeling a fervent desire of gaining information concerning so beneficial a practice, thought it my duty to exert every effort in my power to obtain further instruction; till, in the year 1800, your most excellent work appeared, translated from the English language into German, and published at Leipsic. At the same time I endeavoured to procure genuine vaccine matter as soon as possible; and was at length so fortunate as to succeed, but but with great difficulty, having received some from London, by way of Dantzic.

"Aided by this supply, I commenced my practice the latter end of autumn 1801, by inoculating eight children; and having fully convinced myself of the utility of the operation, and that it was well founded, I carefully preserved matter for the ensuing year.

"In the mean time, M. La Fontaine, a man who has deserved well of the republic of letters, began to print at Warsaw, a work entitled "The Diary of Health;" in which he laboured much to propagate unfavourable opinions concerning my practice; and, as it were, to st angle that most innocent art in its very birth. It is scarcely possible to express, what obstacles and impediments these prejudices of his, which were probably derived from certain authors at Berlin, opposed to the further progress of vaccination. I therefore exerted myself the more diligently and strenuously, in order to eradicate the prejudicial notions on the subject, which prevailed in these kingdoms, from the public mind. Sensible, however, that more dependance was placed on practice than on books and literary altercations, I prepared myself for executing this task, from the 14th of June 1802, through the province, at its own expence; and inoculated 1433 children of the poor with the cow-pock; offering premiums as an encouragement, on account of the mortality of the small-pox, which was then raging on every side.

"For the truth of this statement I appeal to the Warsaw gazettes, in which a testimonial was inserted by the synagogue of the Jews of Tarnoberzeg; where, out of sixty children, whose parents would not suffer them to be vaccinated, twenty died, and two became blind; while out of seventy-one, whom I vaccinated, although they used to eat together, play together, and sleep together, not one died, or received the infection of the small-pox.

"I therefore published, in the Warsaw and Cracow journals, on March 3, 1803, an account of my practice during the year 1802; which produced such an impression on the minds of the people, and particularly those of the lower class, that they eagerly flocked to me with their children, whom they submitted to vaccination. At length, in the year 1803, His Imperial Majesty, by his public letters patent, gave orders for the inoculation of all children throughout the whole kingdom.

"To accomplish this salutary purpose, I undertook one section on my own account; in which I that year inoculated lated 1214 children, and 1314 in the year 1804. The amount of my inoculations for the three years, during the period of only five successive months in each year, beginning in May and ending in October, is 4000 persons; which I have proved to Government by authentic documents, and been so happy as to receive the most gracious marks of its approbation.

"But after all, I must ingenuously confess, that I have nothing so much at heart as to be incorporated with your celebrated institution; into which, I am informed, a foreigner may be elected, provided he has strenuously exerted himself in this cause. I therefore rely on your benevolence for a participation of its honours.—This singular favour I humbly entreat, and shall anxiously expect it by a letter from your own hand.—In the mean while, I transmit to the Society a subscription of five guineas, of which I earnestly solicit their acceptance.

"As the distance of this country, and the inconveniences attending a voyage, are obstacles to my ever seeing you, according to my inclination, I wish at least, Sir, to be in possession of your portrait, if you will be so kind as to present me with it. If, Sir, you would also send, as a token of regard, a bit of cloth of the colour which your are most fond of wearing, I should thankfully receive it; for it is my desire, that those who have displayed the greatest zeal in support of your system, should be dressed in the same uniform on your birthday, and perform an act of public gratitude to the name of Jenner.—That day shall ever be a solemn festival to me, while I exist and enjoy the breath of life.—It now only remains that I bid you adieu, assuring you of the good wishes of,

"Great and illustrious Sir,

"Your most obedient, humble servant,

*Makow, April* 8, 1805. "John Reyss."

I have in my possession a letter from Mr. F. Croese to Dr. Jenner, dated Cheltenham, Sept. 13, 1805, in which he informs him of the introduction of vaccine inoculation at the Cape of Good Hope According to his statement, it was an American, and not a Portuguese vessel, that furnished the inhabitants of the Cape with vaccine virus; which is the more valuable to them, on account of the dreadful ravages of the small-pox in that country.

On the arrival of the vessel, Governor Jansen called a meeting of medical practitioners; by whose persuasions, *together with the encouragement of Government*, the prejudices dices of the people were overcome, and vaccination made a very rapid progress. Every family had their children and their slaves vaccinated. The poor and the slaves were attended gratuitously; and houses were provided by Government for this purpose. Mr. Croese concludes his letter with observing, that he should have been more particular in his inquiries into this subject, had he known that he should ever have had the honour of conversing with a man, who has bestowed such a blessing on mankind.

But while I record with sincere pleasure the successful progress of vaccination in other quarters of the world, and the high degree of estimation in which it is there held, it is a no less grateful task to lay before the public the following extract of a letter, which Dr. Jenner lately received from Dr. Beddoes; which proves that a prophet is not always without honour, even in his own country.

"A common medical friend tells me, that you are informed I am an enemy to the inoculation of the cow-pock instead of the small-pox. *Nothing can be more false.* I believe, I have incidentally contributed more to vaccine inoculation, than any other person in or near Bristol.

"I always recommend to parents, to avail themselves of this security against a formidable disease; *as the greatest service they can render to a young family.* On one or two points, I may perhaps differ in opinion from some of the friends of vaccination; but this in no respect interferes with my opinion, *that it is among the most sacred duties of parents, to insure the lives, senses, and constitutions of their children, against so dangerous an invader as the small-pox, by the practice introduced by you.* What can I add farther, except that *I have had two of my own children inoculated for the cow-pock;* and that *another is to undergo the same process this very day?*

"Make what use you please of these lines, and believe me to be, with great esteem,

Your's, &c.

*Clifton, April* 26, 1806. THOMAS BEDDOES.

To this testimony of a distinguished individual, I have the satisfaction to add that of the Jennerian Society at Plymouth, as expressed in a resolution, at a late meeting of the Society, on the anniversary of Dr. Jenner's birthday. It was as follows.

"At a meeting of the Dock Jennerian Society, it was unanimously resolved, on the report of its medical committee, that having duly considered the evidence and foundation foundation on which the practice of vaccination now rests, and uninfluenced by the late publications which have appeared against it, the Society has great satisfaction in announcing its firm adherence to the opinions which it has uniformly advanced in favour of that practice; and will continue to use its best endeavours, to secure to mankind, the incalculable benefits which may so reasonably be expected from its universal adoption.

JONATHAN ELFORD, President,
RICHARD DUNNING, Secretary.*

I shall conclude this communication with the form of a Testimonial, which, as far as I have hitherto had an opportunity of consulting my medical friends, has met with general approbation. It is calculated to unite the suffrages of those practitioners, who agree in opinion that vaccine inoculation ought to be universally adopted, however they may differ on other points.

*Testimonial in favour of Vaccination, June,* 1806.

WE, the undersigned Fellows and Licentiates of the Royal College of Physicians, and Members of the Royal College of Surgeons, in London, think it our duty thus publicly to express our deep concern at the late mortality occasioned by the small-pox.

The number of victims to this distemper was considerably diminished by vaccine inoculation; but the public confidence in that practice having been shaken by unfavourable reports, circulated with great industry, the inoculation of the small-pox has been in some measure revived, spreading the natural infection, accompanied with its usual ravages.

Diligent inquiry has been made into these reports; and they have in general proved destitute of foundation. We are therefore warranted in concluding, not only from our own experience, but also from the concurrent testimony of other nations, that vaccine inoculation is far preferable to the inoculation of the small-pox; and, if properly conducted, that it is capable of exterminating a disease, which, for many ages, has been the terror and the scourge of mankind.

I am, &c.

JOHN RING.

*New Street, Hanover Square,*
*June* 14, 1806.

P. S. In Mr. Royston's account of cases of secondary small-pox, p. 444. l. 8. for *second,* read *seventh.* This error error of the press is the more necessary to be corrected, because it makes a material alteration in the nature of the case.

